# Construction of ZnO/PCL Antibacterial Coating Potentially for Dental Unit Waterlines

**DOI:** 10.3390/jfb14040225

**Published:** 2023-04-16

**Authors:** Min Xing, Haifeng Zhang, Ling Zhang, Wenhao Qian

**Affiliations:** 1 Shanghai Xuhui District Dental Center, Shanghai 200032, China; xingmin0821@126.com; 2 State Key Laboratory of High Performance Ceramics and Superfine Microstructure, Shanghai Institute of Ceramics, Chinese Academy of Sciences, Shanghai 200050, China; zhanghaifeng@ucas.ac.cn

**Keywords:** polyurethane, dental unit waterlines, ZnO, antibacterial

## Abstract

The formation of bacterial biofilms and the contamination of treatment water within dental unit waterlines can lead to a risk of secondary bacterial infections in immunocompromised patients. Although chemical disinfectants can reduce the contamination of treatment water, they can also cause corrosion damage to dental unit waterlines. Considering the antibacterial effect of ZnO, a ZnO-containing coating was prepared on the surface of polyurethane waterlines using polycaprolactone (PCL) with a good film-forming capacity. The ZnO-containing PCL coating improved the hydrophobicity of polyurethane waterlines, thus inhibiting the adhesion of bacteria. Moreover, the continuous slow release of Zn ions endowed polyurethane waterlines with antibacterial activity, thus effectively preventing the formation of bacterial biofilms. Meanwhile, the ZnO-containing PCL coating had good biocompatibility. The present study suggests that ZnO-containing PCL coating can realize a long-term antibacterial effect on the polyurethane waterlines by itself, providing a novel strategy for the manufacture of autonomous antibacterial dental unit waterlines.

## 1. Introduction

Dental chair units are medical devices used in dental treatment for the examination, diagnosis, and treatment of a variety of dental diseases [1]. Dental unit waterlines are a complex set of interconnected fine-hole waterlines that supply water to all connected irrigating and cooling instruments, cup injection ports, and bowl irrigation outlets. The waterline of the oral comprehensive treatment table is easily polluted during use, and its pipe may form a biofilm due to incomplete cleaning [2]. It has been reported abroad that the bacteria in the waterlines can reach up to 2.0 × 10^5^ CFU/mL within five days after the new oral treatment chair is connected to the waterlines. In the dental chair units, more than 30 kinds of pathogenic microorganisms can be detected in the formed biofilms, and the bacterial concentration in the water outlet of the dental chair units is as high as 10^6^ CFU/mL [3].

Due to the increasingly serious water pollution in dental chair units, the risk of hospital infection in the stomatology department is continually increasing. The study of water pollution in dental chair units and the exploration of its causes have gradually attracted the attention of scholars [4]. Currently, it is recognized that the water pollution in oral comprehensive treatment tables mainly comes from the following three aspects: dental unit waterlines, water sources, and dental mobile phones [5,6,7,8]. The dental unit waterlines have various shapes and numerous interfaces. Meanwhile, the diameter of the pipelines is small and the liquid flow is slow, which leads to the easy adhesion of bacteria and the formation of biofilm on the inside of the lumen. The contamination of the water source makes it difficult to guarantee the quality of the water entering the treatment table. During the use of the dental mobile phone, the drill will come into contact with the blood and saliva of the patient. This will not only pollute the surface of the mobile phone but also suck the dental blood and saliva, which may contain pathogenic microorganisms, back into the cooling water pipe of the mobile phone. In view of the water pollution in the comprehensive treatment table caused by the above factors, the waterways should be cleaned and disinfected regularly to remove bacterial contamination. If necessary, an independent water supply system should be set up to treat the water supply through physical filtration, chemical disinfection, and other methods [9]. However, even if the dental mobile phone and water supply source are effectively disinfected and sterilized, the biofilm formed by the bacteria on the inner wall of the waterway pipeline of the treatment table still exists [10]. To control the water pollution caused by microbial contamination in the waterlines, conventional cleaning and disinfection of the waterlines are used. Irfana Fathima et al. investigated the efficacy of a hypochlorous acid-based disinfectant for disinfecting the dental unit waterlines and found that, compared with the untreated dental unit waterline, no biofilms were observed in the treated dental waterlines [11]. This indicates that hypochlorous acid-based disinfectant can effectively remove the biofilms from the dental unit waterlines. Although disinfection of the waterlines is used, the long-term effectiveness of disinfectants for removing the biofilms on the inner wall of the waterlines still remains a challenge. In addition, the use of disinfectants to disinfect and sterilize waterlines will also cause erosion and reduce the service life of waterlines. Based on this, it is necessary to modify the surface of the inner wall of the waterlines so that it can effectively inhibit the adhesion of bacteria, prevent the formation of biofilms, and reduce the erosion damage of the waterline caused by disinfectants. 

Metal ions, including silver and zinc ions, have a broad spectrum of antibacterial properties and do not produce drug resistance, making them mainstream as inorganic antibacterial agents [12]. Silver-based agents, including Ag^+^ compound agents, silver nanoparticles, and silver coatings, have been commonly used for the decontamination of dental unit waterlines. However, considering sustainability and safety, the large-scale use of silver-based agents is still a challenge [13]. The Zn ion, as one of the essential trace elements of the human body, has multiple biological activities and has the advantages of being environmentally friendly, cheap in price, and having low biological toxicity. In addition, its excellent antibacterial effect and antibacterial mechanism have been confirmed by a large number of studies [14]. Previous studies have shown that Zn-containing biomaterials such as zinc phosphate and zinc polycarboxylate have been employed in oral clinical repair due to their ability to release Zn ions to reduce the growth of the three most common oral pathogens: *Aggregatibacter actinomycetemcomitans*, *Fusobacterium nucleatum*, and *Streptococcus mutans* [15]. Wang et al. deposited ZnO films on a ZrO_2_ dental implant surface and proceeded to anneal them at different temperatures. It was found that ZnO films annealed at 250 °C presented the highest release capability of Zn ions, thus exhibiting the best antibacterial activities against *Streptococcus Sanguinis*, *Bifidobacterium*, and *Porphyromonas Gingivalis* [16]. ZnO was also used as a coating on cotton fabric to realize the long-term antimicrobial performance of textiles [17]. Furthermore, a synergistic effect of ZnO and other materials was also utilized to obtain the superior antibacterial activity of ZnO-based composite materials. Zhang et al. fabricated a ZnO-loaded graphene oxide nanocomplex (ZnO-GO) and favorable antibacterial performance against *Escherichia coli* (*E. coli*) and *Staphylococcus aureus* (*S. aureus*) was achieved through the synergistic effect of GO and ZnO [18]. Synergistic hybrid materials including N-halamine polymers (PAM-Cl) and ZnO were used to fabricate a microfiber-based antibacterial platform using an electrospinning technique. PAM-Cl/ZnO hybrid materials inherited the antibacterial activities of PAM-Cl and ZnO. They presented enhanced synergistic antibacterial properties against *E. coli* and *S. aureus* based on the multimodal bactericidal effects, including the contact killing of PAM-Cl and the release killing of Zn^2+^ [19]. Moreover, the synergistic antibacterial effect of ZnO and polymorphonuclear neutrophils was also obtained. ZnO not only demonstrated antibacterial activity but also enhanced the antibacterial performance of polymorphonuclear neutrophils by promoting bacterial phagocytosis efficiency, proinflammatory cytokine expression, and reactive oxygen species production [20].

Based on the superior antibacterial activity of ZnO, ZnO-based antibacterial coatings have been widely used for biomedical applications [21]. However, as far as we know, the study of fabricating ZnO coatings on dental unit waterlines to inhibit bacterial adhesion and prevent the formation of biofilms has not been reported. Therefore, in the present work, we innovatively introduced ZnO to the surface of polyurethane (PU) waterlines using polycaprolactone (PCL) with a good film-forming capacity. On the one hand, the ZnO-containing PCL coating improved the hydrophobicity of waterlines, thus inhibiting the adhesion of bacteria. On the other hand, the continuous slow release of Zn ions endowed the waterlines with antibacterial activity, effectively preventing the formation of bacterial biofilms, which can prevent and control hospital infection in the department of stomatology.

## 2. Materials and Methods

### 2.1. Sample Preparation

Polyurethane (PU) with a diameter of 12 mm and a thickness of 1 mm was utilized as a substrate material in this work. To fabricate a zinc oxide coating on the surface of PU, 0.5 g of polycaprolactone (PCL, Shanghai Aladdin Biochemical Technology Co., Ltd., Shanghai, China) was immersed in 10 mL of dichloromethane under magnetic stirring at 400 rpm, then 0.1, 0.3, and 0.5 g of zinc oxide (30 ± 10 nm, Shanghai Macklin Biochemical Technology Co., Ltd., Shanghai, China) were separately added into the as-prepared PCL-containing dichloromethane solution. Subsequently, PU plates were immersed in the solution with different contents of zinc oxide for 5 s, then pulled out using dip-coating technology at a speed of 50 μm/s and dried in a drying oven at 60 °C for 12 h. The dipping process was conducted one time. The corresponding samples were denoted as ZnO1-PCL, ZnO3-PCL, and ZnO5-PCL, each with different contents of ZnO. In contrast, a PCL sample without ZnO was also fabricated using the same method.

### 2.2. Surface Characterization

The surface morphologies of PU, PCL, ZnO1-PCL, ZnO3-PCL, and ZnO5-PCL were observed using a tungsten filament scanning electron microscope (SEM, S-3400 N, Hitachi, Japan) with an acceleration voltage of 15 kV. The coating thicknesses were detected by observing the cross-section using the SEM. X-ray diffraction (XRD, D2 phase, Bruker, Karlsruhe, Germany) using Cu Ka as the radiation source was used to investigate the phase compositions of PU, PCL, ZnO1-PCL, ZnO3-PCL, and ZnO5-PCL. The contact angle instrument (SL200B, Solon, Shanghai, China) was applied to evaluate the surface wettability of PU, PCL, ZnO1-PCL, ZnO3-PCL, and ZnO5-PCL. To investigate the ion release behavior, ZnO1-PCL, ZnO3-PCL, and ZnO5-PCL samples with a surface area of ~3 cm^2^ were separately immersed in 10 mL of 0.9% NaCl aqueous solution for 1, 4, and 7 days. The ion release amount was detected using inductively coupled plasma atomic emission spectroscopy (ICP-AES, 5100, Agilent, Santa Clara, CA, USA). To determine the amount of ZnO on the coatings of sample surfaces, ZnO1-PCL, ZnO3-PCL, and ZnO5-PCL were separately immersed in 10 mL of 0.9% NaCl solution with 0.1 M hydrochloric acid at 80 °C for 48 h, and the total concentration of Zn ions was detected by ICP-AES.

### 2.3. Antibacterial Properties Evaluation In Vitro

Gram-negative *Escherichia coli* (*E. coli*, ATCC 25922, provided by China General Microbiological Culture Collection Center) and Gram-positive *Staphylococcus aureus* (*S. aureus*, ATCC 25923, provided by China General Microbiological Culture Collection Center) were used to investigate the antibacterial properties of PU, PCL, ZnO1-PCL, ZnO3-PCL, and ZnO5-PCL. In detail, 1 mL bacterial suspensions with a concentration of 1.0 × 10^7^ CFU/mL were separately co-cultured with PU, PCL, ZnO1-PCL, ZnO3-PCL, and ZnO5-PCL for 2 h and 24 h at 37 °C. For the agar plate count, *E. coli* or *S. aureus* on the surface of PU, PCL, ZnO1-PCL, ZnO3-PCL, and ZnO5-PCL were collected and diluted tenfold with sterile 0.9% NaCl solution. Then, 100 μL of the diluted bacterial solution was introduced onto the agar culture plate and cultured for another 18 h. Subsequently, images of the agar culture plates were taken and the bacterial colonies on the agar culture plates were counted. The antibacterial rate with the reduction in bacterial percentage was calculated according to the following equation [22]: R = (Nc − Ne)/Nc·100%, where Nc and Ne are the numbers of bacterial colonies in the control group and experimental group, respectively. To observe the bacterial morphology using the SEM, *E. coli* or *S. aureus* on the surface of PU, PCL, ZnO1-PCL, ZnO3-PCL, and ZnO5-PCL were fixed with a 4% paraformaldehyde (PFA) solution for 30 min, and then the bacteria were dehydrated with gradient ethanol solutions of 30%, 50%, 75%, 90%, 95%, and 100% (*v*/*v*). Finally, the bacterial morphologies were observed using the SEM at an accelerating voltage of 15 kV, and the corresponding photographs were taken.

### 2.4. Cell Compatibility Assessment

Mouse fibroblast cells (L929, provided by Cell Bank, Chinese Academy of Sciences, Shanghai, China) were used to investigate the biocompatibility of PU, PCL, ZnO1-PCL, ZnO3-PCL, and ZnO5-PCL. First, PU, PCL, ZnO1-PCL, ZnO3-PCL, and ZnO5-PCL were immersed in 3 mL of α-MEM cell medium for 12 h and 24 h, and the extraction solution was obtained. Then, L929 cells with a density of 1.0 × 10^4^ cells/mL were seeded in a 96-well transparent plate for 24 h at 37 °C in a 5% CO_2_ incubator. Next, extraction solutions were added to replace the original medium and the cells were cultured for another 24 h. For the cell live/dead staining, 20 μL PBS of calcein-AM (5 μM) and propidium iodide (2 μM) were introduced and cultured for another 15 min. Then the cell morphologies were observed using a fluorescent microscope (Olympus, Tokyo, Japan) and the corresponding images were taken. To assess cell viability, 120 μL α-MEM of 10 *v*/*v* % alamarblue was introduced and cultured for another 2 h. Finally, 100 μL of the medium was transferred into a 96-well black plate, and the fluorescence intensity of the reduced alamarblue was measured using an excitation wavelength of 560 nm and an emission wavelength of 590 nm. Thus, the fluorescence intensity was proportional to the cell viability.

### 2.5. Statistical Analysis

The data were expressed as the mean ± standard deviation. The statistical significance of the difference was analyzed using multiple *t* tests (and nonparametric tests)—one per row. Statistical analysis was assessed using the GraphPad Prism 6 software. A value of *p* < 0.05 indicated a statistically significant difference.

## 3. Results and Discussion

### 3.1. Surface Characterization

**Figure 1:**
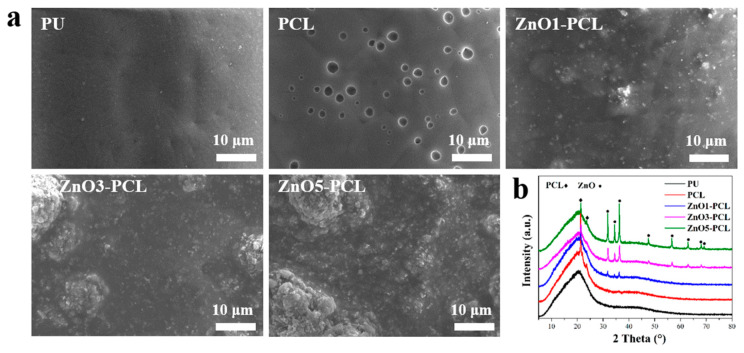
(**a**) SEM surface morphologies of PU, PCL, ZnO1-PCL, ZnO3-PCL, and ZnO5-PCL; (**b**) XRD spectra of PU, PCL, ZnO1-PCL, ZnO3-PCL, and ZnO5-PCL.

Bacterial biofilm in dental unit waterlines is a widespread problem and leads to a potential risk of infection for both dental staff and patients [23]. Traditional disinfection methods cannot effectively remove the bacterial biofilm from the dental unit waterlines; even worse, the use of disinfectants will cause the waterlines to erode and reduce their service life. To solve this problem, a ZnO-containing PCL coating was prepared on the PU waterlines. The surface morphologies of PU, PCL, ZnO1-PCL, ZnO3-PCL, and ZnO5-PCL are shown in Figure 1a. PU presents a relatively smooth surface, while holes can be seen in PCL, which may be attributed to the volatilization of dichloromethane. Particles of ZnO can be observed in ZnO1-PCL, and this becomes more obvious with the increasing contents of ZnO during the preparation process. Figure 1b shows the XRD patterns of PU, PCL, ZnO1-PCL, ZnO3-PCL, and ZnO5-PCL. Characteristic peaks of ZnO [24] appear on ZnO1-PCL, ZnO3-PCL, and ZnO5-PCL, and the peak intensity increases with the addition of ZnO content. This suggests that a ZnO coating was successfully fabricated on the surface of PU. To assist in the fabrication of the ZnO coating, PCL was added, and the feature peak was also detected, as presented in Figure 1b.

**Figure 2:**
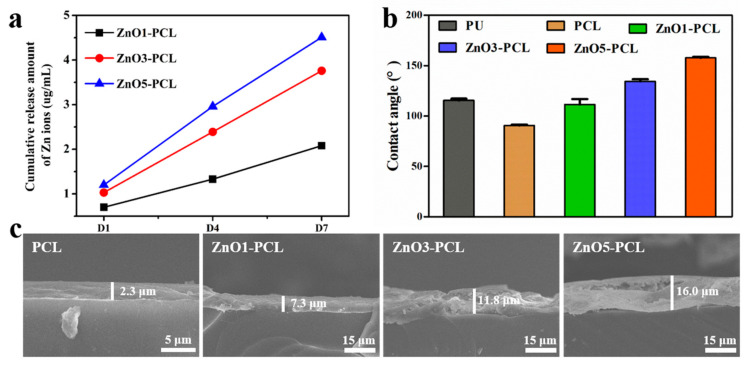
(**a**) Cumulative release amount of Zn ions in ZnO1-PCL, ZnO3-PCL, and ZnO5-PCL at 1, 4, and 7 days; (**b**) Water contact angles of PU, PCL, ZnO1-PCL, ZnO3-PCL, and ZnO5-PCL; (**c**) Coating thickness detected by observing the cross-section using the SEM.

To confirm that the coating we prepared on the inner surface of the waterlines can release Zn ions and achieve the long-term slow release of Zn ions, the Zn ion release behaviors of ZnO1-PCL, ZnO3-PCL, and ZnO5-PCL were investigated, and the results are presented in Figure 2a. The release amount of Zn ions from ZnO1-PCL, ZnO3-PCL, and ZnO5-PCL is closely related to the Zn ion concentration during the preparation process. ZnO5-PCL shows the highest release amount of Zn ions, followed by ZnO3-PCL and ZnO1-PCL. The release amounts of Zn ions within 7 days from ZnO1-PCL, ZnO3-PCL, and ZnO5-PCL were 2.1, 3.8, and 4.5 μg/mL, respectively. The total contents of ZnO from ZnO1-PCL, ZnO3-PCL, and ZnO5-PCL were determined by dissolving the ZnO coating in a hydrochloric acid-containing sodium chloride solution, and the total concentration of Zn ions was detected by ICP-AES. As presented in Table 1, the total concentrations of Zn ions in ZnO1-PCL, ZnO3-PCL, and ZnO5-PCL are 28.4, 82.2, and 147.8 μg/mL. Based on the release rate and total contents of Zn ions from the sample surface, the results imply that ZnO-containing PCL coatings can control the long-term sustained release of Zn ions. 

Surface wettability plays a great role in inhibiting bacterial adhesion [25]. Therefore, the water contact angles of PU, PCL, ZnO1-PCL, ZnO3-PCL, and ZnO5-PCL were detected, and the results are presented in Figure 2b. The average water contact angles of PU, PCL, ZnO1-PCL, ZnO3-PCL, and ZnO5-PCL are 115.5°, 90.6°, 111.5°, 134.4°, and 157.7°, respectively. PU shows a hydrophobic surface with a water contact angle above 90°. Compared with PU, the water contact angle of PCL is slightly reduced. With the addition of ZnO, the water contact angle increases. ZnO5-PCL presents the highest water contact angle value, followed by ZnO3-PCL and ZnO1-PCL. This indicates that a more hydrophobic surface can be obtained by increasing the content of ZnO in the ZnO-containing PCL coating. The coating thicknesses were detected by observing the cross-section using the SEM, and the results are presented in Figure 2c. The coating thicknesses of PCL, ZnO1-PCL, ZnO3-PCL, and ZnO5-PCL are 2.3 μm, 7.3 μm, 11.8 μm, and 16.0μm, respectively.

### 3.2. Antibacterial Activity In Vitro

**Figure 3:**
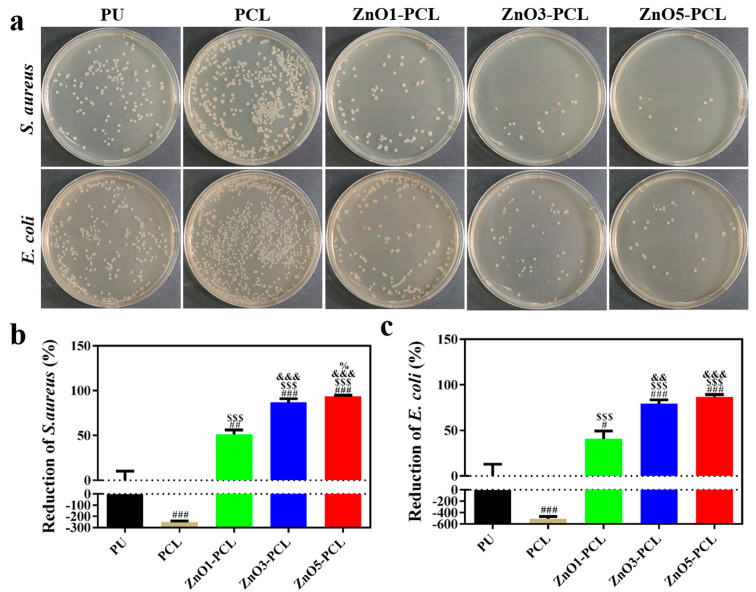
(**a**) Photographs of re-cultivated *S. aureus* and *E. coli* colonies on agar culture plates of PU, PCL, ZnO1-PCL, ZnO3-PCL, and ZnO5-PCL after the co-culture of bacteria and various samples for 2 h and the corresponding antibacterial rates of PU, PCL, ZnO1-PCL, ZnO3-PCL, and ZnO5-PCL against *S. aureus* (**b**) and *E. coli* (**c**) based on the plate counting method. # *p* < 0.05, ## *p* < 0.01, ### *p* < 0.001 vs. PU; $$$ *p* < 0.001 vs. PCL; && *p* < 0.01, &&& *p* < 0.001 vs. ZnO1-PCL; % *p* < 0.05 vs. ZnO3-PCL.

**Figure 4:**
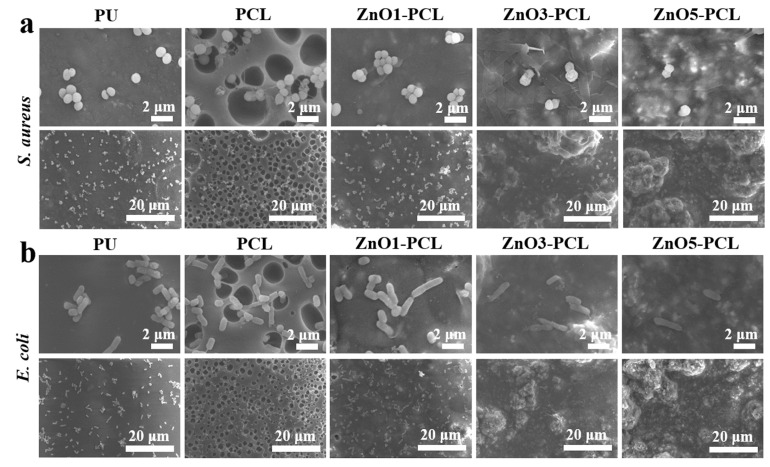
SEM bacteria morphology of *S. aureus* (**a**) and *E. coli* (**b**) cultured with PU, PCL, ZnO1-PCL, ZnO3-PCL, and ZnO5-PCL for 2 h.

Gram-negative *E. coli* and Gram-positive *S. aureus* were chosen as bacteria strains to investigate the antibacterial activity of PU, PCL, ZnO1-PCL, ZnO3-PCL, and ZnO5-PCL. To investigate the bacterial adhesion in the early stage, bacteria were co-cultured with PU, PCL, ZnO1-PCL, ZnO3-PCL, and ZnO5-PCL for 2 h. Figure 3a shows the photographs of the bacterial colonies of *S. aureus* and *E. coli* on PU, PCL, ZnO1-PCL, ZnO3-PCL, and ZnO5-PCL. Compared with PU, the number of bacterial colonies adhering to PCL increases, while it decreases on ZnO1-PCL, ZnO3-PCL, and ZnO5-PCL. This indicates that PCL can promote bacterial adhesion in the early stage, which may be attributed to the holes in the sample surface. As presented in Figure 3b,c, the reductions of *S. aureus* on ZnO1-PCL, ZnO3-PCL, and ZnO5-PCL are 51.1%, 87.1%, and 93.9%, respectively. Similarly, the reductions of *E. coli* on ZnO1-PCL, ZnO3-PCL, and ZnO5-PCL are 40.8%, 79.6%, and 86.7%, respectively. This suggests that ZnO5-PCL exhibits the highest rate of bacteriostasis, followed by ZnO3-PCL and ZnO1-PCL. From the SEM bacteria morphology presented in Figure 4, it can be seen that compared with PU, lots of bacteria adhered to the holes in the PCL sample surface, while the bacteria amount reduced on the ZnO-containing PCL samples and the bacteria membranes were deformed to some extent, especially the bacteria on ZnO5-PCL. 

**Figure 5:**
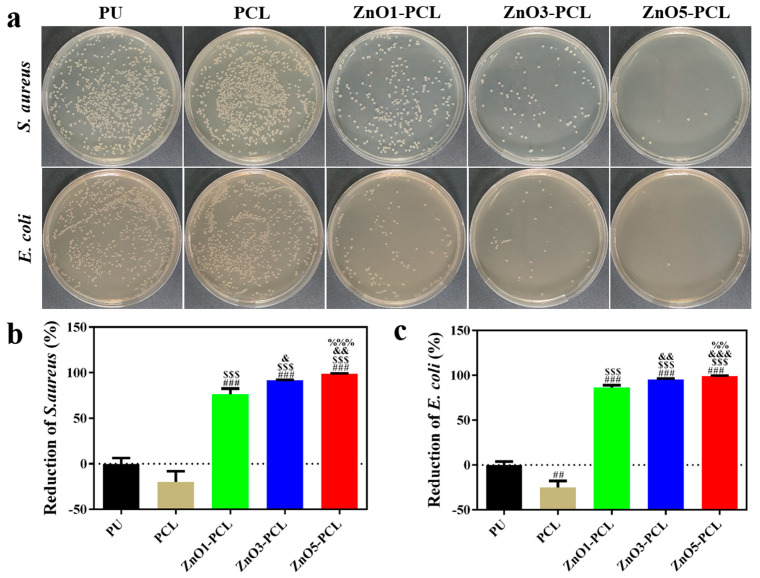
(**a**) Photographs of re-cultivated *S. aureus* and *E. coli* colonies on agar culture plates of PU, PCL, ZnO1-PCL, ZnO3-PCL, and ZnO5-PCL after the co-culture of bacteria and various samples for 24 h and the corresponding antibacterial rates of PU, PCL, ZnO1-PCL, ZnO3-PCL, and ZnO5-PCL against *S. aureus* (**b**) and *E. coli* (**c**) based on the plate counting method. ## *p* < 0.01, ### *p* < 0.001 vs. PU; $$$ *p* < 0.001 vs. PCL; & *p* < 0.05, && *p* < 0.01, &&& *p* < 0.001 vs. ZnO1-PCL; %% *p* < 0.01, %%% *p* < 0.001 vs. ZnO3-PCL.

**Figure 6:**
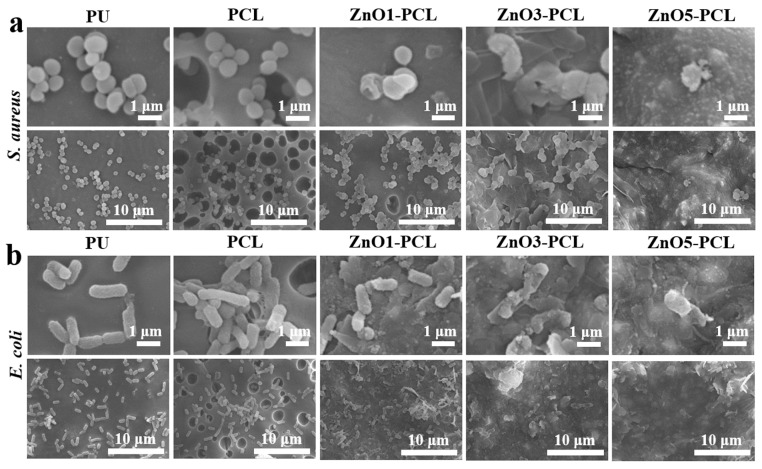
SEM bacteria morphology of *S. aureus* (**a**) and *E. coli* (**b**) cultured with PU, PCL, ZnO1-PCL, ZnO3-PCL, and ZnO5-PCL for 24 h.

To investigate the relatively long-term antibacterial activity of PU, PCL, ZnO1-PCL, ZnO3-PCL, and ZnO5-PCL, bacteria were co-cultured with PU, PCL, ZnO1-PCL, ZnO3-PCL, and ZnO5-PCL for 24 h. As presented in Figure 5a, lots of bacteria colonies can be observed on PU and PCL. Compared with PU, the number of bacteria colonies on the ZnO-containing PCL samples is reduced, especially on ZnO5-PCL. As shown in Figure 5b,c, ZnO5-PCL shows the highest antibacterial rate against *S. aureus* at 99.0%, followed by ZnO3-PCL with an antibacterial rate of 91.6%, and ZnO1-PCL with an antibacterial rate of 76.3%. Similarly, the antibacterial rates of ZnO1-PCL, ZnO3-PCL, and ZnO5-PCL against *E. coli* are 86.5%, 95.3%, and 99.2%, respectively. Among them, ZnO5-PCL displays the highest antibacterial rate. The SEM bacterial morphologies in Figure 6 further confirm this point; the bacteria amount on the ZnO-containing PCL samples is visibly reduced and the bacteria membranes are seriously deformed, especially on ZnO5-PCL.

### 3.3. Cell Compatibility In Vitro

**Figure 7:**
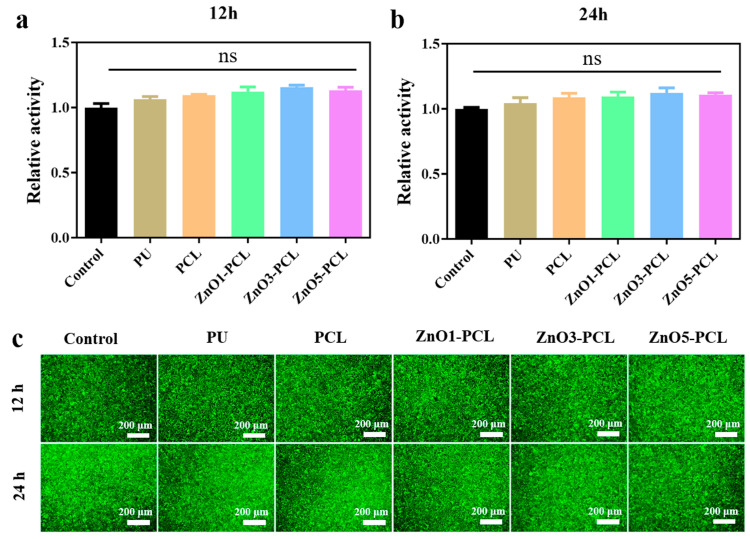
Cell viability of L929 cells co-cultured with extraction solution of PCL, ZnO1-PCL, ZnO3-PCL, and ZnO5-PCL for 24 h with immersion time of 12 h (**a**) and 24 h (**b**); Live/dead staining of L929 cells co-cultured with extraction solution of PCL, ZnO1-PCL, ZnO3-PCL, and ZnO5-PCL for 24 h with immersion time of 12 h and 24 h (**c**). The “ns” indicates no significant difference.

Since water flows directly into the patient’s mouth after passing through the dental unit waterlines, it is important to assess the biosafety of the surface coating of waterlines. L929 cells were used to evaluate the cell compatibility of the extraction solution of PU, PCL, ZnO1-PCL, ZnO3-PCL, and ZnO5-PCL with different immersing times. Cell viability and cell live/dead staining were performed, and the results are shown in Figure 7. There is no significant difference in cell viability among them, as presented in Figure 7a,b. This indicates that ZnO-containing PCL coatings have good cell compatibility. The cell live/dead staining results further confirm this, where lots of live cells with green fluorescence are visible, and dead cells with red fluorescence are barely visible. This suggests that ZnO-containing PCL coatings fabricated on the surface of PU waterlines have good biocompatibility and biosafety.

Dental unit waterlines play an important role in supplying dental chair units with therapeutic water. The waterlines are commonly made of soft plastics such as polyurethane, with a small inner diameter which is conducive to bacterial adhesion, leading to the formation of biofilms [26]. Biofilms containing microbial communities embedded in a self-produced extracellular matrix are more resistant to disinfectants [27]. Therefore, biofilms formed on the inner walls of waterlines are one of the main sources of water pollution. To control the water pollution caused by microbial contamination in the waterlines, conventional cleaning and disinfection are used. Zhu et al. investigated the effect of chlorine-containing disinfectants on dental unit waterline contamination control and found that while chlorine-containing disinfectants could reduce water pollution, they could not remove biofilms [28]. The effectiveness of sodium hypochlorite regarding contamination control of the output water from dental unit waterlines is variable. Karpay et al. reported that 5.25 percent NaClO diluted at 1:10 achieved the proposed American Dental Association goal of fewer than 200 CFU/mL [29]. However, bacterial counts above 500 CFU/mL were found when dental unit waterlines were treated with 0.31% NaClO [30]. Bacterial contamination was still found in dental unit waterlines when treated with 0.12% chlorhexidine gluconate and 0.02% hydrogen peroxide every week [31,32]. This may be attributed to the fact that microorganisms produce resistance against chemical disinfectants or that different chemical disinfectants have a selection for specific microorganisms. Moreover, chemical disinfectants may have adverse effects, such as causing the erosion of waterlines, the formation of foam, and the staining of dental equipment [33]. Therefore, herbal disinfectants such as aloe vera were also used to replace chemical disinfectants when treating microbial contamination in the dental unit waterlines [34]. Silver-based disinfectants are among the commonly used agents for the decontamination of dental unit waterlines, including silver compound agents and silver nanoparticles, and silver itself can inhibit bacterial growth. Furthermore, hybrid materials containing silver ions and hydrogen peroxide are commonly used due to their synergistic effects. However, considering sustainability and safety, the large-scale use of silver-based agents is controversial [13]. Up until now, most studies have focused on the disinfection of dental unit waterlines. The application of material coatings to dental unit waterline tubing to inhibit bacterial adhesion and prevent the formation of biofilms is seldom reported. When dental unit waterlines are in an unused and static state, there is moisture on the inner surface and this moisture can easily lead to bacterial infection. Therefore, in the present work, we fabricated a ZnO-containing PCL coating on the dental unit waterlines. A ZnO-containing PCL coating displayed satisfactory antibacterial activity which could inhibit early bacterial adhesion and prevent the formation of bacterial biofilms. The promising antibacterial activity of the ZnO-containing PCL coating may be attributed to the hydrophobic surface and released zinc ions. Furthermore, ZnO-containing PCL coatings presented no cytotoxicity to L929 cells and showed good cell compatibility. Based on these findings, ZnO-containing PCL coating on dental unit waterlines may suppress the proliferation and adhesion of bacteria and may prevent and control hospital infection in the department of stomatology.

## 4. Conclusions

In summary, a ZnO-containing PCL composite coating was applied to the surface of PU waterlines. The ZnO-containing PCL composite coating includes a hydrophobic surface with a contact angle of up to 157.7°, which can inhibit initial bacterial adhesion. The reduction in the initial bacterial adhesion of *S. aureus* and *E. coli* on ZnO5-PCL was up to 93.9% and 86.7%, respectively. Moreover, ZnO-containing PCL composite coatings on PU waterlines exhibit a slow and sustained release of Zn ions which present good antibacterial activity against *E. coli* and *S. aureus*. The antibacterial rates of ZnO5-PCL against *S. aureus* and *E. coli* were 99.0% and 99.2%, respectively, which effectively inhibited the formation of bacterial biofilms. Meanwhile, the composite coating presents good biocompatibility. This work provides a novel strategy for the design of antibacterial dental unit waterlines by constructing a ZnO-containing PCL composite coating that exhibits good antibacterial activity and may prevent and control hospital infection in the department of stomatology.

## Figures and Tables

**Table 1:** Total concentration of Zn ions dissolved from ZnO-PCL coating on different sample surfaces.

Samples	Zn Ions Concentration (μg/mL)
ZnO1-PCL	28.4
ZnO3-PCL	82.2
ZnO5-PCL	147.8

## Data Availability

The data presented in this study are available on request from the corresponding author.
